# Evaluation of outcomes and utility of abdominal aortic aneurysm surveillance in octogenarians and nonagenarians

**DOI:** 10.1308/rcsann.2023.0089

**Published:** 2023-12-01

**Authors:** IT Nasir, SS Shoab, MG Bani-Hani

**Affiliations:** Lancashire Teaching Hospitals NHS Foundation Trust, UK

**Keywords:** Aneurysm, Surveillance, Screening, Octogenarian

## Abstract

**Introduction:**

The aim of this study was to evaluate the utility of our regional abdominal aortic aneurysm (AAA) screening programme in octogenarians and nonagenarians. This was to help decide whether discontinuation might be appropriate in certain instances. Primary outcomes were the number of patients who reached threshold (5.5cm) and the number where intervention was offered. Secondary outcome was cost effectiveness.

**Methods:**

A retrospective review of a regional AAA surveillance database was carried out to evaluate outcomes. Data collected included patient age, sex, date of first and last scan, initial and latest size of aneurysm, outcome, time under surveillance and total number of scans. Patients were divided into three groups (80–84 years, 85–89 years and 90+ years).

**Results:**

The number of patients in this age group was 354. Only 2.0% (*n*=7) of patients underwent intervention. Threshold size was achieved in 8.3% (*n*=18), 14.8% (*n*=18) and 26.7% (*n*=4), in the age groups 80–84 years, 85–89 years and 90+ years, respectively. Of these patients, operative intervention was possible in 2.8% (*n*=6), 0.8% (*n*=1) and 0% (*n*=0), respectively.

**Conclusion:**

A relatively small number of octogenarians and nonagenarians reach the threshold size during surveillance. An even smaller proportion require repair of their aneurysm. While there may be a role for AAA surveillance in octogenarians in highly selected groups, these data should inform the discussions made with individual patients. It should also inform future evaluation of such surveillance.

## Introduction

Despite an ageing population, there are relatively limited data on abdominal aortic aneurysm (AAA) surveillance in octogenarians and nonagenarians. The UK National Health Service Abdominal Aortic Aneurysm Screening Programme (NAAASP) was set up in 2009 based on trials including patients aged 65–79 years. Patients with aneurysms below the 5.5cm threshold are followed up at intervals. The programme discharged the patients if the AAA remained under 4cm after 15 annual scans (at the age of 80). The protocols for further follow-up in smaller aneurysms are not uniform and there is relatively little evidence to support any of these.

Sweden, Western Australia, Denmark, Norway, New Zealand, Italy and the USA also have their own screening AAA programmes.^1^ All programmes agree on the definition of 30mm or above being considered aneurysmal; however, depending on the measured diameter, there are differences in the surveillance interval between programmes. For example, in the USA, three-yearly scans are offered if the size is 30–34mm and six-monthly if 45–54mm; however, in the UK, this is yearly and three-monthly, respectively. All programmes screen men only, although New Zealand, Italy and the USA also screen women.

The age at which patients are invited for screening also varies among countries. The UK, Norway and Sweden invite patients in their 65th year, Italy 65 years and over, Western Australia 65–79 years, Denmark 65–74 years, New Zealand, if patients have high cardiovascular risk, and the USA 60–75+ years with a history of tobacco use or first-degree relative with an AAA.

### Current literature on AAA surveillance in octogenarians and nonagenarians

There are three studies published in the last three years regarding surveillance of AAA in octa- and nonagenarians from the UK,^2^ Canada^3^ and Spain.^4^ The first study, carried out in the UK, investigated outcomes (management of threshold aneurysm, aneurysm-related and all-cause mortality) of patients aged 85 years or above to identify whether discontinuation of surveillance might be safe. There was a total of 101 patients (88 male) who were stratified according to initial aneurysm diameter (<40mm, 40–50mm or >50mm). Median follow-up time was 56.0 months. Most patients (72.3%) undergoing surveillance did not reach threshold. Only 1 patient in the <40mm group reached threshold compared with 5 (11.6%) and 22 (75.9%) in the 40–50mm and >50mm groups, respectively (*p*<0.0001). Of the 28 patients who reached threshold, only 8 (7.9% of the total number) underwent repair. These findings suggested discontinuation of aneurysm surveillance in patients aged 85 or above with aneurysms <40mm may be done with an acceptable risk. In patients with aneurysms approaching threshold, early assessment of fitness for surgery may prevent unnecessary surveillance.

The second study, from Canada, explored the use of ultrasound surveillance in detecting AAAs reaching threshold in those aged 80 years or above versus those aged <80 years, incidence of AAA repair and cost–benefit analysis of surveillance. Old age and small enrolment aneurysm size were significantly protective against AAA growth. Patients >80 years were less likely to reach threshold for repair, compared with the younger population (adjusted hazard ratio, 0.77; 95% CI 0.61–0.97). They were also substantially less likely to undergo elective AAA repair (adjusted hazard ratio, 0.34; 95% CI 0.24–0.47). In terms of cost–benefit analysis, for every octogenarian with an initial AAA size between 3.0 and 3.9cm who received elective repair, 51 patients were enrolled without elective repair. This corresponded to an estimated extra spending of $33,139 in ultrasound fees.

The third study, from Spain, aimed to study the natural history of small (<55mm) AAAs in octogenarians and nonagenarians to assess the need for follow-up and/or invasive treatment. They enrolled 310 patients (256 male), divided into three groups based on aneurysm size (30–39mm, 40–49mm and 50–54mm). Median follow-up was 37.9 months. Overall 62 (20%) AAAs reached the threshold for surgical repair. However, only eight of these were repaired electively (12.9%; 2.6% of the total). They also found AAA size <40mm was an independent protective factor from rupture (HR 0.13; 95% CI 0.03–0.48), reaching threshold for repair (HR 0.08; 95% CI 0.04–0.16) and death (HR 0.63; 95% CI 0.42–0.95). The authors concluded that conservative management would be sensible in this age group, with strict selection of the patients who would benefit from eventual repair.

## Methods

The PubMed database was searched to identify relevant articles. The keywords ‘abdominal aortic aneurysm’, ‘surveillance’ in ‘octogenarians’, ‘nonagenarians’ and ‘octogenarians and nonagenarians’ were utilised. This generated 116, 19 and 6 articles, respectively. All abstracts were screened for outcomes related to reaching threshold and/or performing intervention or cost analysis in octogenarians and nonagenarians.

We conducted a retrospective review of our regional AAA surveillance database. This is a prospectively maintained database. Data collected included patient age, sex, date of first scan (or when the aneurysm was first known), initial size of aneurysm, date of the latest scan, size of aneurysm, outcome, time under surveillance and total number of scans. Patients were followed up until their latest scan, date of discharge or date of death.

We included those patients aged 80 or above at the time of the initial scan (or when their aneurysm was first known). We did not include patients that were detected through the NAAASP. These were considered a different cohort in view of the earlier age of their diagnosis. We defined a minimum time on surveillance/number of scans required for patients to be included. This was >six months (minimum two scans) for aneurysms >4.5cm and >one year (minimum one scan) for aneurysms <4.5 cm in accordance with the UK national screening programme recommendations.

We excluded non-AAA aneurysms (*n*=47), those who were at threshold on initial scan (*n*=16) and those with inadequate follow-up (*n*=45). Other exclusions were aortic ulcer (*n*=1), patients who moved out of area (*n*=2) and patients with incomplete data (*n*=2) (Table 1).

**Table 1:** Exclusion criteria

Type of aneurysm	Frequency (*n*)
Non-AAA aneurysms	47
5.5cm AAA on initial scan	16
Abdominal aortic ulceration	1
Moved out of area	2
Missing data	2
Inadequate follow-up	45

Primary outcomes were the number of patients reaching threshold and/or having aneurysm repair. Secondary outcome was cost implications by collecting data on the number of scans performed. Patients’ outcomes were analysed by age group (80–84 years, 85–89 years and 90 years or above) to try to identify an age-specific threshold if possible (Table 2).

**Table 2:** Characteristics and outcomes of included patients

	Overall, *n*=354	80–<85 years, *n*=217 (61.3%)	85–<90 years, *n*=122 (34.5%)	90+ years, *n*=15 (4.2%)
Age at referral (years)	83.8 (80–100)	81.8 (80–84)	86.5(85–89)	92.0 (90–100)
Current age (years)	88.2 (81–106)	86.2 (81–100)	90.6 (86–99)	97.0 (91–103)
Sex	M: 268 (75.7%) F: 86 (24.3%)	M: 162 (74.7%) F: 55 (25.3%)	M: 93 (76.2%) F: 29 (23.8%)	M: 13 (86.7%) F: 2 (13.3%)
Reached threshold (5.5cm)	40 (11.3%)	18 (8.3%)	18 (14.8%)	4 (26.7%)
Had intervention	7 (2.0%)	6 (2.8%)	1 (0.8%)	0 (0%)
Total number of scans	1564 (4.4)	952 (4.4)	557 (4.6)	55 (3.7)
Time on surveillance (years)	3.72 (0.42–14.75)	3.86 (0.58–14.75)	3.52 (0.42–11.00)	3.27 (1.17–8.17)

Continuous variables are shown as mean (range/average) and categorical variables as number (percentage): M = male; F = female

## Results

Out of 824 on surveillance in our region, 354 patients (43%) were eligible for inclusion (Table 2). Only 2.0% (*n*=7) of patients underwent surgical intervention in our cohort, with the presenting aneurysm size ranging between 3.6cm and 5.4cm.

As an arterial vascular centre, we inherited the local surveillance programmes from the merging units in the area. Interestingly, 28 patients (7.7%) were originally referred with subaneurysmal aortas (<3cm) but continued to have surveillance scans. Of these, only 3 were discharged appropriately, 8 died while on surveillance (28.6%) and 17 (60.7%%) remained on surveillance. They had a total of 94 scans performed during this period. The average time on surveillance was 4.3 years. It is often the case that once a patient is placed on surveillance, the decision is not reviewed unless the threshold is reached. Throughout the surveillance period 72 patients (20.3%) were discharged from surveillance. Around one-quarter of discharges were requested either by the patients or their attorney. Overall 28.2% (*n*=100) died during surveillance; 15% of the patients were clinically discharged as unfit for intervention.

### 80–84 years

There were 217 patients in this group. Only 18 (8.3%) of the patients reached threshold and 6 patients underwent aneurysm repair (2.8%). The smallest initial aneurysm size that proceeded to intervention was 3.6cm in this subgroup. None of the patients who started with an aneurysm size <3.5cm had any intervention.

### 85–89 years

There were 122 patients in this group. Of these, 18 patients (14.8%) reached the threshold for repair, though only one patient went on to have surgical intervention (0.8%) (this patient died 10 months after EVAR was performed from non-AAA causes). In this patient, the initial aneurysm diameter was 5.2cm. All patients in this group who started with an aneurysm size <4.0cm did not proceed to have any intervention.

### 90+ years

Only 15 patients in our cohort were aged 90 or above, as most of the nonagenarians are excluded already through their local practice or the patient’s preference. Unsurprisingly, a larger percentage reached threshold size in this group (*n*=4, 26.7%). None of them proceeded to have intervention.

### Cost implications

Based on the data collected, we estimated the number of potential unnecessary scans in specific subgroups of patients who would not progress to have any intervention in their lifetime. These subgroups included patients aged 80 years or above with an initial diameter <3.5cm, those aged 85 or above starting at <4.0cm and all patients aged 90 or above regardless of aneurysm size. Excluding these patients may have saved 318, 232 and 55 scans, respectively, in addition to 94 scans in subthreshold aortas (<3.0cm) making a total of 699 unnecessary scans. These patients would have other overhead expenses (e.g. hospital transport and CT scans when limited or inconclusive US assessment).

## Discussion

Many studies confirm that it is possible to have acceptable outcomes in octogenarians and nonagenarians from operative intervention for AAA. This is for those who are suitable for intervention. However, relatively few are found to be suitable for intervention.

It has also been calculated that lengthier intervals between surveillance scans may be an acceptable option. This may include scans at two-yearly intervals (even three-yearly scans have been proposed). Longer scanning intervals are relevant especially to aneurysms measuring between 3 and 3.9cm. The authors calculated that in a cohort of 285,000 patients, changing from annual to biannual scans in smaller aneurysms may translate into a saving of £580,000 over a course of 30 years. On the other hand, scanning at even longer intervals may mean unacceptably higher rates of rupture and aneurysm-related deaths.^5^ Since even longer interval scanning has cost implications, it is reasonable to remove the patients from surveillance who are clearly not going to benefit. This study looked at just the cost of the scan. Other overheads such as transport, secretarial costs and the surgeon’s input in interpreting results are not included.

Our data support and confirm the published results from other studies. Only a small proportion of octogenarians and nonagenarians reach threshold size of their aneurysm, and an even smaller percentage are fit for intervention at that stage. Interestingly, an initial aneurysm size of <40mm in those aged 80 or above has been suggested in all three studies as a possible way to stratify AAA surveillance in this age group. These results from studies that took place in the UK, Canada and Spain seem to be congruous and generally applicable.

Considering the proportion of patients reaching threshold size, eventual intervention thresholds for discontinuation of surveillance may be inferred in the different age groups. In our population, all patients aged 80–84 with initial diameter <3.5cm, aged 85–89 with <4.0cm and all aged 90 or above (regardless of initial aneurysm size) may potentially have their surveillance discontinued if acceptable after due consultation. Therefore, we submit that our study is important in informing individual discussion with patients. (These data may also help make policy decisions regarding this group of patients.)

Rather than discontinuing surveillance altogether, increasing scan intervals may be considered. This may serve the purpose of reassurance and still make some savings. There is some usefulness of surveillance in providing reassurance to certain patients, and therefore continuation of such even in older patients may become a matter of personal preference. For others, knowing the exact size of the aneurysm may be required for the purpose of insurance and/or driving.

Our findings also showed a downward trend in the time on surveillance and total number of scans had by each age subgroup. Those aged 80–84 years were on surveillance for longer and had more scans compared with those aged 90 or above. This is likely to be due to the higher mortality rate in nonagenarians in general (it may also reflect a more liberal approach in discharging nonagenarians from surveillance).

An external review for the UK National Screening Committee (NSC) in 2016 found that in patients who had subaneurysmal aortas (2.5–2.9cm) at 65 years, 8.3% would reach large (>5.4cm) aneurysm size at a mean time of 13.2 years follow-up.^6–9^ Though this was related to those aged 65 years, if applied theoretically to our population of 80 years and above, they are expected to reach threshold around the age of 93 when they are unlikely to be offered an intervention.^10–12^ The life expectancy in the North West of England is 77.9 and 81.7 for males and females, respectively.^13^ The mean age of patients at referral was 83.9 and mean current age was 87.3. Despite an estimated lower number of octogenarians in our region compared with the national average, around 40% of those on AAA surveillance in our cohort were in that age group. Where the proportion of octogenarians is different in other parts of the UK, a higher life expectancy may mean that the number of unnecessary scans is likely to be even greater.

With each advancing age subgroup, the proportion of patients who reached threshold increased. This provides evidence that the age at which patients reach clinically relevant aneurysms is increasing. This is in keeping with previous reports.^14,15^ The NAAASP design aimed to discharge the patients after the age of 80 (consistent with findings in our region). However, there is a cohort of ‘fitter’ octogenarians that may benefit from continuing surveillance. Stricter selection criteria may be required to be applied in this high-risk group.

The potential benefits to individual AAA screening units need to be considered and balanced with the disadvantages/difficulties associated with the implementation of any change in surveillance. In a cost effectiveness analysis, Marshall *et al*^5^ acknowledge that although the long-term cumulative cost savings may be high for the programme, realising the benefits of minimised surveillance strategies for individual screening units is likely to be difficult. The reduction in the total number of surveillance scans for each unit is likely to be too small to allow a reduction in overall staffing. Similarly, the cost savings per AAA screening unit will be relatively small and unlikely to release funds for other activities. Whether the cost savings from refining surveillance strategies justifies a change in clinical practice has yet to be determined.

However, it is appreciated that the reduction may allow improvements in the ease of scheduling or free staff for other activities such as continuous professional development, quality improvement, audit and research. The psychological impact of either extending the intervals in surveillance or stopping surveillance completely has not yet been studied properly. Despite these factors, subjecting elderly patients to a regular surveillance program at least behoves us as caring health professionals to discuss frankly with them the likely benefit of any surveillance offered. While some may choose to stay and be reassured, others may well decide to leave the programme. Indeed, a quarter or more of the patients where surveillance was discontinued was because of patient choice in our study.

More work is needed across all regions to investigate a more ‘efficient’ system for AAA surveillance in octogenarians. Factors such as a current diagnosis of cancer, dementia, frailty score and current nursing home residence, for example, would all be relevant. Early exclusion of the EVAR 2 category of unfit patients would be vital.^16^ A tailored individual approach with at least one face-to-face review at a specified time point in the surveillance programme in octogenarians would also improve the cost-effectiveness of such a programme.

We believe this work will help to inform the decision-making process regarding continuing surveillance. Any advice offered may not necessarily be prescriptive, but rather explaining these findings may help the patient to reach a reasonable decision.

## Conclusion

There are a significant number of octogenarians and nonagenarians on local AAA surveillance programmes. Once enrolled, there are no checks and/or filter mechanisms to re-visit. It is right that AAA surveillance in some octogenarians can be continued. Our data suggest that this may benefit only selected cases. Offering surveillance to all comers, however, may not offer any advantage for many patients.

It is possible that AAA surveillance in this population may be stratified by age group as well as initial aneurysm size. For the present, discussions with patients should be informed by studies such as the present one. Discussions may include both continuation (or otherwise) of surveillance as well as the intervals at which surveillance is done. This would make allowances for individual patient concerns and reassurance issues. An agreed mutual decision-making process in an individual manner should continue until further studies and data are available.
